# Retrospective panoramic radiographic evaluation of acute leukemia patients with fractal analysis

**DOI:** 10.1186/s12903-025-06625-8

**Published:** 2025-07-26

**Authors:** Hüseyin Balbay, Serdar Uysal

**Affiliations:** Faculty of Dentistry, Department of Dentomaxillofacial Radiology, Hacettepe University, Kalaba mahallesi 30. Sokak No: 5 VM Medical Park, Ankara Hastanesi Keçiören, Ankara, Turkey

**Keywords:** Fractal analysis, Panoramic image, Acute leukemia, Trabecular structure

## Abstract

**Background:**

The aim of this study is to examine the changes in the trabecular structure of the jaw bones in acute leukemia (AL), which can cause extensive osteopenia using the Fractal Analysis (FA).

**Methods:**

Panoramic images (PI) of 45 patients with AL who did not have any additional disease affecting the bone constituted the case group. PI of 45 patients without any disease affecting the bone tissue were used for the control group. For each PI, a total of 4 regions of interest (ROI) located on two anterior and two posterior regions of the mandible were determined. FA was applied to the determined ROIs and the results were analyzed statistically.

**Results:**

FA values ​​of the case group were significantly lower than those of the control group. With respect to gender, only the posterior FA values ​​of the females were significantly lower than those of the males, in the case group. There was no correlation between age, blood values ​​and FA values.

**Conclusions:**

The changes in the trabecular structure of the bone due to the effects of AL can be detected by the FA. Gender and age have no effect on FA values, and there is no correlation between blood values ​​and FA values. AL causes extensive osteopenia and therefore reduced trabecular complexity, as the trabecular jawbone has a high bone turnover rate.

**Supplementary Information:**

The online version contains supplementary material available at 10.1186/s12903-025-06625-8.

## Introduction

Diseases affecting internal organs can also present with findings in the dental structures. In some cases, dentists may be the first to suspect a systemic disease based on early signs observed in the oral cavity. Oral structures such as the teeth, jaws, oral mucosa, and temporomandibular joint may be affected due to metabolic, endocrine, or vascular disorders. Leukemia, a significant category of blood diseases, involves malignant anomalies in hematopoiesis. When normal hematopoiesis is disrupted, a series of mutations occur in early hematopoietic precursors, preventing their progeny from maturing properly while allowing uncontrolled proliferation. These malignant cells eventually enter the bloodstream and spread throughout the body [1, 2].

Although the classification of leukemia is complex, it is clinically divided into acute and chronic forms. Acute leukemia (AL) is particularly significant as it is the most common malignancy in childhood. The disease progresses rapidly and, without treatment, often has a poor prognosis. Radiographically, extensive osteopenia of the bones can be seen in AL. Jaw involvement is more common in areas of developing teeth and typically manifests as rarefied osteitis in the periapical regions. Radiographic features include poorly defined, patchy radiolucent areas and, in some cases, onion-skin-like periosteal new bone formation. Destruction of the cortical border of the lamina dura and follicular structures may occur, and the direction or position of erupting teeth may change [1, 2].

Panoramic imaging (PI) is a two-dimensional diagnostic tool commonly used as an initial imaging method for evaluating large areas of the jaws. PI is a simple, practical, and cost-effective technique. Beyond the assessment of dental structures, bone morphology can also be evaluated, and various analyses can be performed using different measurement techniques [3–5]. PI has great potential, particularly in the early detection of large-area trabecular pattern changes [4–6].

Various methods are used to analyze dental tissues. Fractal analysis (FA) is a statistical texture analysis technique based on fractal mathematics, used to identify complex structural patterns. It measures the self-similarity and complexity of a structure. FA has been applied to describe shapes—such as curves, points, and surfaces—that cannot be effectively represented by conventional geometry but are self-similar at different scales [6]. Numerous studies have demonstrated its usefulness in analyzing biological images [3, 7–10]. While FA has certain limitations, it offers a quantitative method to detect changes in bone structure. It is unaffected by variables such as projection geometry or radiation dose, and it is a non-invasive, accessible, and cost-effective method. As a result, its use in medicine and dentistry continues to grow [11–13]. Its validity and reliability have been reported in many dental studies, especially in evaluating bone structure. Many studies have explored the use of FA to detect osteoporotic changes in the jaw bones associated with metabolic diseases [10, 15–21].

The image of the internal structure of the alveolar bone resembles a lattice formed by thin spicules, trabeculae and lamellae. Trabecular bone features a branching structure that exhibits fractal characteristics, such as self-similarity and scale invariance. Therefore, measuring fractal dimensions (FD) using fractal geometry can help assess trabecular complexity and bone structure [14]. This allows information on subtle, otherwise invisible, changes in the three-dimensional trabecular bone to be obtained from two-dimensional images using FA values [15]. Various pathological conditions may alter the morphology of anatomical structures, and these changes can be detected using fractal parameters [12, 16].

The aim of our study was to examine changes in the trabecular structure of jaw bones affected by AL using FA.

## Materials and methods

The patients who referred to the Hacettepe University, Faculty of Dentistry, Department of Dentomaxillofacial Radiology, Ankara, Turkey between January 2015 and March 2020 were included in the study.

The inclusion criteria of the patients in the study were as follows:

- Diagnosed with AL,
- Availability of a panoramic radiographic image,
- No systemic disease affecting bone metabolism such as osteoporosis, osteopetrosis, hyperthyroidism, hypothyroidism, hyperparathyroidism, hypoparathyroidism, renal osteodystrophy, Cushing’s syndrome, hypophosphatasia, rickets, osteomalacia, sickle cell anemia, thalassemia. Besides, the patients with a systemic disorder affecting bone metabolism were excluded. Patients diagnosed with AL whose blood values were available were included in the study. Hemoglobin (gr/dl), erythrocyte (x10^6/µL), leukocytes (x10^3/µL), lymphocyte (x10^3/µL), platelets (x10^3/µL), calcium (mg/dl), phosphorus (mg/dl), uric Acid (mg/dl) values were recorded. Patients diagnosed with AL apply for dentistry with a request for consultation before AL treatment is started.

**Fig. 1:**
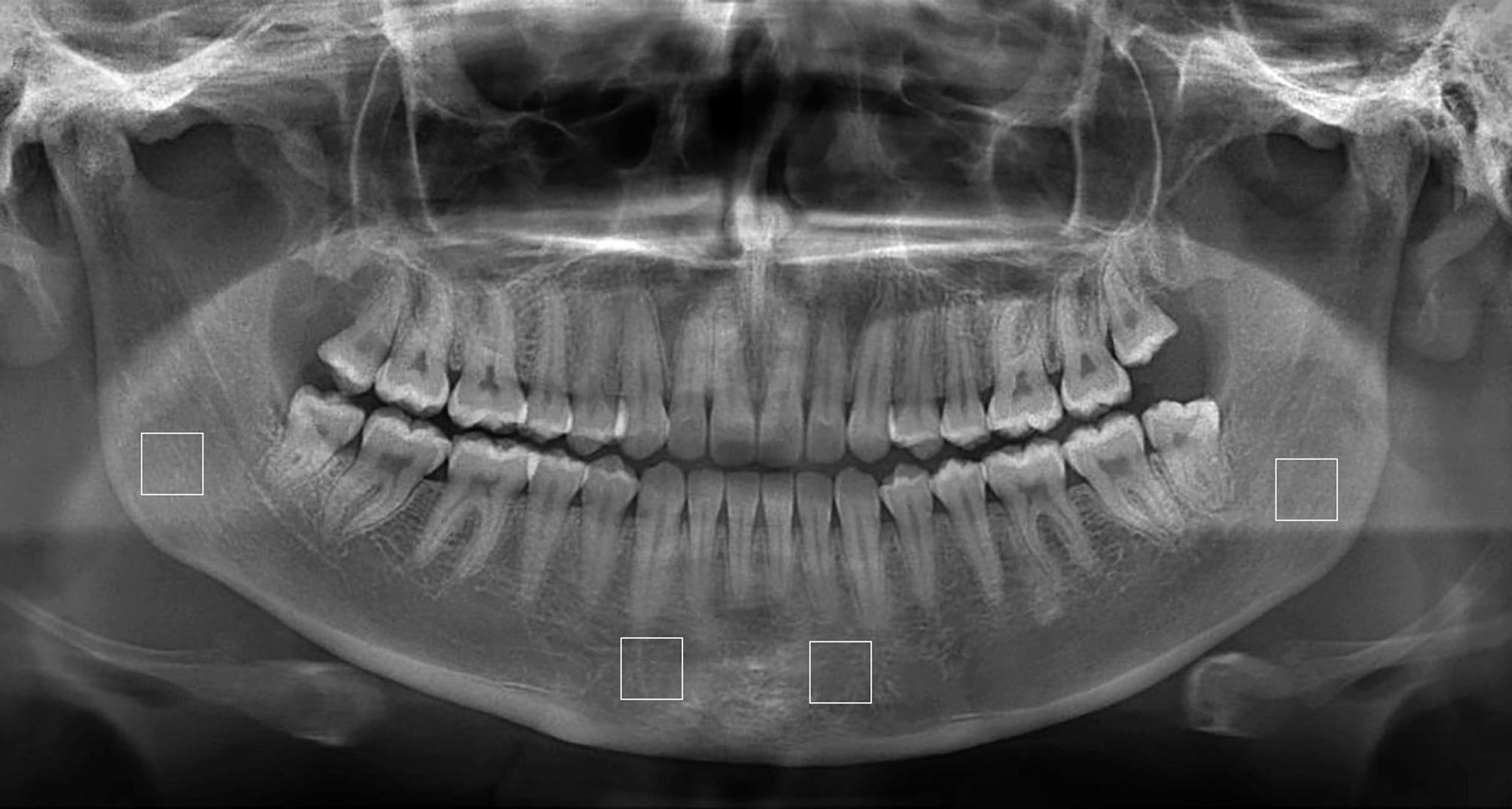
Obtaining ROIs on a panoramic radiograph

From 80 patients diagnosed with AL, 45 patients (20 females, 25 males) constituted the case group depending on the availability of the panoramic radiograph and absence of any systemic disease affecting bone metabolism. The control group consisted of 45 healthy individuals (25 females, 20 males) and had panoramic radiographs. All panoramic images included in the study were digitally obtained using Veraview IC5 (Morita Corporation, Japan) device with 60–70 kVp, 1-7.5 mA, 5.5–10 s. The device’s ideal panoramic radiography acquisition technique was used during the exposure. Image processing and analysis were performed by using Image J software (Image J 1.47u; NIH, Maryland, MD). In each panoramic radiograph four Regions of Interests (ROIs), two in the right and left anterior region and two in the right and left posterior region, were determined. The ROIs were manually placed because it was important that no anatomical structure with the potential to influence the FA value was located within the ROI. ROIs were selected by a single observer and inter- or intra-observer reliability was not assessed. It was ensured that in those regions there were no anatomical formations such as the lamina dura, periodontal ligament, alveolar crest, tooth root surface, mandibular canal and basis mandible. 50 × 50 pixel ROIs were created in the anterior and posterior regions (Fig. 1). Anterior Region Fractal Analysis (ARFA) was created by taking the average of the FA values of the Anterior Region right and left ROIs in each panoramic image. Likewise, Posterior Region Fractal Analysis (PRFA) was created by taking the average of the FA values of the right and left ROIs of the posterior region.

**Fig. 2:**
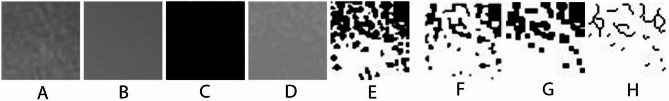
Application of fractal analysis, **A**: ROI, **B**: remove fine and medium brightness variations, **C**: blurred image subtracted from the original, **D**: pixel value of 128 added to standardize brightness, **E**: binarized using a threshold of 128 Gy levels, **F**: binarized using a threshold of 128 Gy levels, **G**: the binary image dilated, **H**: binary image skeletonized

The FA was applied as described previously [10–13]. In detail, all obtained ROIs were cropped (Fig. 2A) and duplicated. The duplicated ROIs were blurred using a Gaussian filter (kernel size = 35) to remove subtle and median variations in image brightness (Fig. 2B). The blurred images were subtracted from the original image (Fig. 2C) and a value of 128 was added to each pixel to obtain an image with a mean value of 128 (Fig. 2D). The resulting image was binarized with a threshold of 128 grey values (Fig. 2E). In order to reduce the noise in the image, it was first eroded once (Fig. 2F) and then dilated once (Fig. 2G). After dilation, the binary image was outlined and skeletonized (Fig. 2H). Finally, the resulting image was used for FA. In the skeletonized binary images, skeletonized structures indicate bone structure, while non-skeletonized structures indicate bone marrow. The fractal dimension of the skeletonized image was calculated by the box counting method. Equal sized gridded squares were overlaid on the image and the number of tiles with trabecular bone was determined. The widths of the squares were 2, 3, 4, 6, 8, 12, 16, 32 and 64 pixels. The number of counted tiles was then plotted against the total number of tiles on a double logarithmic scale. Finally, FD was calculated from the slope of the graph.

FD values of the case group and the control group were compared statistically. It was also analyzed whether there was a relationship between the FD values of the case group in relation to age, gender and blood values belonging to the AL diagnosis period. Normal distribution of numerical variables was analyzed by Shapiro-Wilk test. The difference between the case and control groups was evaluated by independent samples t-test for normally distributed FA values and Mann-Whitney U test for non-normally distributed FA values. Spearman correlation coefficient was used to analyze whether there was a relationship between FA values and ROIs, and between the biochemical values of the patients and FA values. The minimum sample size required to determine a correlation between FA values and biochemical values of AL patients with an error of 0.05 and power of 0.80 was calculated as 34 (G*Power 3.1.9).

## Results

The case group was between the ages of 15–69 and the control group was in the age range of 15–68 years. The mean age of the study group was 38.9, and the mean age of the control group was 34.9 (Table 1).

**Table 1:** Age distribution of patient and control groups

GROUP	GENDER	NUMBER	MEAN	SD	MIN	MAX
**PATIENT**	Female	20	39,05	15,936	15	69
	Male	25	38,88	14,740	18	68
	Total	45	38,96	15,106	15	69
**CONTROL**	Female	25	30,08	11,394	15	68
	Male	20	41,10	15,400	17	55
	Total	45	34,98	14,279	15	68

SD: Standart deviation, min: minimum, max: maximum

The ARFA value was found to be 1.056 ± 0.102 for the case group and 1.193 ± 0.088 for the control group. The value of the PRFA in each panoramic image included in the study was calculated as 0.803 ± 0.147 for the case group and 1.034 ± 0.128 for the control group (Table 2). Both ARFA and PRFA values were statistically lower in the case group compared to the control group (*p* < 0.05).

**Table 2:** Fractal analysis values of anterior and posterior areas

		MEAN	SD	MIN	MAX
**ARFA Values**	PATIENT	1,056	0,102	0,761	1,231
	CONTROL	1,193	0,088	1,021	1,344
	P	< 0,001			
**PRFA Values**	PATIENT	0,803	0,147	0,524	1,057
	CONTROL	1,034	0,128	0,527	1,282
	P	< 0,001			

SD: Standart Deviation, Min: Minimum, Max: Maximum

The ARFA value was 1.076 ± 0.094 and 1.213 ± 0.079 for the females in the case and control groups, respectively. The value was 1.038 ± 0.106 and 1.166 ± 0.093 for the males in the case and control groups, respectively.

The PRFA value of the females was 0.754 ± 0.148 in the case group and 1.050 ± 0.100 in the control group. Regarding males, it was measured as 0.841 ± 0.136 in the case group and 1.014 ± 0.155 in the control group (Table 3). When both ARFA and PRFA values were evaluated according to gender, no statistical difference was found in both the control group and the case group, except for PRFA values (*p* > 0.05).

**Table 3:** Fractal analysis values of anterior and posterior regions by gender

			MEAN		SD		MIN		MAX
**ARFA Values**	**PATIENT**	F	1,076		0,094		0,911		1,231
		M	1,038		0,106		0,761		1,206
		T	1,056		0,102		0,761		1,231
		P	= 0,263						
	**CONTROL**	F	1,213		0,079		1,052		1,337
		M	1,166		0,093		1,021		1,344
		T	1,193		0,088		1,021		1,344
		P	= 0,087						
**PRFA Values**	**PATIENT**	F	0,754		0,148		0,524		1,057
		M	0,841		0,136		0,560		1,053
		T	0,803		0,147		0,524		1,057
		P	= 0,025						
	**CONTROL**	F	1,050		0,100		0,808		1,209
		M	1,014		0,155		0,527		1,282
		T	1,034		0,128		0,527		1,282
		P	= 0,343						

SD: Standart deviation, min: minimum, max: maximum, F: female, M: male

When the correlation values of blood biochemistry values and FA values of the case group were analyzed, it was observed that there was only a moderate negative correlation between ARFA and lymphocyte values (Table 4). Otherwise, no correlation was found between blood biochemistry values and FA values.

**Table 4:** Correlation values of blood values of all patients at the time of diagnosis and ARFA and PRFA measurements

	ARFA			PRFA		
	rho	*p*	*n*	rho	*p*	*n*
**Age**	0,145	0,342	45	-0,159	0,298	45
**Hemoglobin (gr/dl)**	0,038	0,803	45	-0,221	0,145	45
**Erythrocyte(x10^6/µL)**	-0,037	0,808	45	-0,198	0,191	45
**Leukocytes (x10^3/µL)**	-0,165	0,28	45	0,165	0,279	45
**Lymphocyte (x10^3/µl)**	-,481**	0,001	45	0,276	0,066	45
**Platelets (x10^3/µl)**	0,042	0,785	45	0,004	0,979	45
**Calcium(mg/dl)**	-0,009	0,951	45	0,104	0,496	45
**Phosphorus (mg/dl)**	-0,05	0,745	45	0,129	0,398	45
**Uric Acid(mg/dl)**	-0,264	0,079	45	0,219	0,148	45

** Correlation is significant at the 0.01 level (2-tailed)

## Discussion

The FA has been utilized across various scientific disciplines to analyze the fundamental components of images that lack standard geometric shapes and cannot be described using Euclidean geometry. These structures exhibit complexity and self-similarity across different scales. Numerous researchers have demonstrated the utility of FA in analyzing the complex, self-repeating patterns found in biological images [3, 7–9, 17–19].

It has been reported that changes in the architecture of cancellous bone in postmenopausal women can be assessed using FA [20]. Radiographic FD of the alveolar bone correlates significantly with bone density, with FD values increasing as bone density rises [21]. In dentistry, In dentistry, FA has been shown to quantitatively detect changes in the alveolar bone [22] and in the trabecular architecture of the mandibular condyle in patients with temporomandibular joint disorders [23].

Trabecular bone exhibits a branching pattern that reflects fractal characteristics such as self-similarity and a lack of a well-defined scale. Therefore, applying fractal geometry to radiographs and measuring FD provides valuable insight into trabecular complexity and bone architecture. In the literature, higher FD values are associated with increased structural complexity, while lower FD values correspond to simpler trabecular patterns [7, 24, 25, 26]. The assessment of trabecular bone, which is more metabolically active and exhibits a higher turnover rate than cortical bone, offers valuable diagnostic insights. Consequently, trabecular bone provides a more sensitive indicator for detecting changes in bone structure [13]. The assessment of trabecular bone, which is more metabolically active and exhibits a higher turnover rate than cortical bone, offers valuable diagnostic insights. Consequently, trabecular bone provides a more sensitive indicator for detecting changes in bone structure [27–30]. Accordingly, panoramic radiographs were utilized in our study to apply FA over broader areas with larger ROIs.

In AL and other hematologic malignancies, the proliferation of malignant or abnormal cells results in impaired production of erythrocytes, leukocytes, and platelets, leading to clinical manifestations such as anemia and thrombocytopenia [31, 32]. Sickle cell anemia, for instance, causes reduced trabecular bone volume and an osteoporotic radiographic appearance, similar to AL. Demirbaş et al. [33] investigated changes in mandibular trabecular bone structure using a single ROI on panoramic radiographs in sickle cell anemia patients. They reported lower FA values in the disease group compared to controls, consistent with our findings. Their study found mean FA values of 1.6855 in the disease group and 1.7196 in the control group. In contrast, our study found mean anterior region FA (ARFA) values of 1.056 in the AL group and 1.193 in controls, while mean posterior region FA (PRFA) values were 0.803 and 1.034, respectively.

Several studies have explored the applicability of FA in evaluating bone resorption in osteoporotic jaw bones. Oliveira et al. [34], using panoramic radiographs, reported lower FD values in osteoporotic patients compared to controls, with mean FA values of 1.36 (right posterior) and 1.35 (left posterior) in the patient group, and 1.41 and 1.40, respectively, in the control group. However, some studies report conflicting results. Demiralp et al. [27], analyzed trabecular patterns via FA on panoramic images of cancer patients and healthy individuals undergoing bisphosphonate therapy. Using the box-counting method and four ROIs of 18 × 19 pixels, they observed no significant differences between groups: mean FD values were slightly higher in the study group (1.39) than controls (1.38), but the difference was not statistically significant (*p* ≥ 0.05). These findings could be attributed to trabecular sclerosis, a hallmark of bisphosphonate-related osteonecrosis [35, 36] as bisphosphonates are known to cause extensive bone sclerosis [27].

Guleç et al. [37] investigated the impact of bruxism on mandibular trabecular bone using digital panoramic radiographs. They selected ROI sizes of 50 × 50 pixels for the condyle and 100 × 100 pixels for other mandibular regions. While FA values were generally lower in bruxist patients, only the right condyle showed a statistically significant difference. Reported mean FA values in bruxism patients were 1.38 (right dentate) and 1.37 (left dentate). Though similar in methodology, the differences in ROI placement and bruxism diagnosis—based only on clinical observation and patient history—may account for discrepancies with our study. The authors [37] also suggested that variations in occlusal habits and bite force could explain unilateral differences in FA results.

Sex-based differences in FD values have also been explored. Demiralp et al. [27] and Arsan et al. [23] reported lower FA values in females on both sides, though these differences were not statistically significant. No consistent relationship was found between age and FA values. Similarly, Güleç et al. [37] found lower FD values in females but no significant age correlation. In line with these studies, our results showed lower FA values in females than males in the posterior region, while anterior values were comparable. No significant association between FA and age was observed in our data.

Additionally, Arsan et al. [23] studied FA in patients with chronic renal failure. Using ROIs of 35 × 30 pixels from the region between the left second premolar and first molar on panoramic images, they evaluated correlations between FD values and biochemical markers (PTH, ALP, Vitamin D, Ca, P). Consistent with our findings, no significant associations were identified.

As no previous studies have examined AL patients using FA, direct comparisons regarding sample size or findings were not feasible. Given the retrospective nature of our study and its reliance on patients diagnosed with AL and imaged at a single center within a specific time frame, the sample size is relatively limited compared to large-scale studies. Therefore, findings should be interpreted with caution in light of this limitation.

Furthermore, the age difference between case and control groups and the unbalanced gender distribution are important considerations. These factors stem from the retrospective design and limited availability of AL patients who had undergone panoramic imaging, and should be considered when evaluating our results.

This study demonstrates that the differences in FA values between AL patients and healthy controls are statistically significant. These results suggest that dentistry and hematology can work synergistically in patient care. Dentists, by recognizing reduced bone complexity or early signs of osteopenia in panoramic radiographs using FA, may contribute to the early detection of AL and facilitate timely referrals to hematologists.

## Conclusions

AL leads to extensive osteopenia and a reduction in trabecular bone complexity. FA is a practical and effective method for assessing trabecular bone architecture and detecting structural changes induced by AL. In patients with AL, FD values decrease due to the loss of trabecular bone elements.

To our knowledge, this is the first study to compare FA-based assessments of bone structure with biochemical parameters in AL patients. We believe that our findings may serve as a foundation for future, more comprehensive studies aimed at quantitatively tracking trabecular changes throughout the progression of the disease.

One limitation of this study is its retrospective design and relatively small sample size, which should be considered when interpreting the results. A second limitation is the absence of biochemical data for the control group. Due to the retrospective nature of the study, blood tests were not conducted in healthy controls, as it would have been ethically inappropriate to collect unnecessary blood samples. As a result, a direct comparison of biochemical parameters between groups was not possible, which limits our ability to fully interpret the systemic effects of AL. Besides, the study does not account for key biological confounders such as age-related bone loss or hormonal changes (e.g., postmenopausal status in women), which could influence the outcomes that should be considered.

Further prospective studies with larger sample sizes and complete biochemical data sets are necessary to validate and expand upon these findings.

## Electronic supplementary material

Below is the link to the electronic supplementary material.

Supplementary Material 1

## Data Availability

The datasets used and/or analysed during the current study are available from the corresponding author on reasonable request.

## Abbreviations

AL: Acute Leukemia
PI: Panoramic Imaging
FA: Fractal Analysis
FD: Fractal Dimension
ROI: Regions of Interest
ARFA: Anterior Region Fractal Analysis
PRFA: Posterior Region Fractal Analysis
