# Supplementary material for: Retrospective panoramic radiographic evaluation of acute leukemia patients with fractal analysis
Source: BMC Oral Health. 2025 Jul 26;25:1260. doi: 10.1186/s12903-025-06625-8 (PMC12296621; doi:10.1186/s12903-025-06625-8)
Supplement: Supplementary file 1 — Supplementary Material 1 [file 12903_2025_6625_MOESM1_ESM.pdf]

CONSENT FORM

Name and Surname: Date, Time of Application:
File No:
Gender:

PERSON TO CONTACT IN CASE OF EMERGENCY
Name and surname:
Phone:

INFORMATION

Dear patient/attorney/legal representative;

Please read the following explanations carefully. This “Patient Information and Consent Form” has been prepared for our patients who will be examined in Hacettepe University Faculty of Dentistry Oral, Dental and Maxillofacial Radiology Clinics and voluntarily participate in scientific studies. Our patients are required to inform their physicians about their current systemic diseases, medications and general health status. The responsibility arising from your concealment or failure to declare any subject belongs to you. In the Oral, Dentomaxillofacial Radiology Clinic, a detailed examination of the head, skin, eyes, ears, nose, lymph nodes, paranasal sinuses, chewing muscles, jaw joints, salivary glands, soft tissues such as gums, tongue, cheeks, teeth and bone structures in the oral cavity is performed in line with your complaint, medical and dental history. If there is no preventive condition (such as pregnancy), additional dental x-rays may be requested if necessary. As a result of clinical and radiologic evaluation, your diagnosis is made and you are referred to the relevant clinics.

The aim of radiologic evaluation is to diagnose pathologies and diseases in the teeth and/or bone and/or soft tissue that cannot be seen visually. Imaging examinations of your teeth, jaw and face are necessary for the diagnosis of conditions that may affect your general or oral health, treatment planning and follow-up of treatment stages. Intraoral (periapical, occlusal, bitewing) and extraoral digital radiographs (panoramic, joint X-rays) and cone beam computed tomography are performed in our clinic using X-rays. Your physician determines the number and type of X-rays to be ordered. The X-rays used in X-rays are harmful to living tissue. For this reason, your physician will order the minimum number of X-rays required for your diagnosis, treatment planning, follow-up of your treatment stages and control purposes.

If you are pregnant or suspected of being pregnant, it is your obligation to inform your doctor and the X-ray staff. In this case, radiographs will be requested if necessary and you will be dressed in a lead apron and the minimum number of X-rays will be taken.

For educational and scientific purposes, photographs, X-rays or video images may be taken, and clinical and radiological data may be used for scientific, educational or research purposes, with the patient's identity information kept confidential.

If the patient is under the age of 18, his/her mother/father or legal representative must sign the Patient Consent Form.

During your application to our clinic, it is your most natural right to be informed about the examination and evaluation, examinations, procedures and costs to be performed before dental treatment. After learning the benefits and possible risks of the treatment and procedures, it is up to your decision to approve the procedure.
Please read the explanations below about these imaging techniques and other examinations to be performed in our clinic.

INSPECTIONS

- Digital radiography (periapical, bitewing, occlusal, panoramic, joint X-rays)

The aim of radiological evaluation is to diagnose pathologies in teeth, bone and soft tissues that cannot be seen on examination. X-rays of the mouth, jaw or joint area may be required at the beginning of treatment, during treatment and after treatment for control purposes. Removable prostheses (dentures) in the mouth should be

removed before intraoral x-rays and all metal objects in the head and neck area (earrings, hairpins, necklaces, glasses, piercings, removable prostheses, dentures, hearing aids, etc.) should be removed before extraoral x-rays. Instead of traditional films, an imaging method is used in which x-ray sensitive sensors are used and the image is transferred to a computer and monitored. During intraoral X-rays, phosphor plates placed in a disposable plastic bag are used. Without radiologic examination, the pathology and its cause may not be accurately determined and the success of the treatment cannot be evaluated. If you are pregnant or suspect pregnancy, it is your obligation to inform your physician and the X-ray staff. Nausea and sometimes vomiting may occur during X-rays. You must remain immobile during the procedure. If you move or if the phosphor plate is shifted, the image may be distorted and a retake may be necessary.

Radiographs:

- They can show areas of decay that are not visible on visual examination (for example, decay between teeth) and decay or other problems that develop under existing fillings.

- Provides information about bone loss due to gum disease.

- It allows visualization of problems in the root canal.

- It is useful and necessary in implant preparation and placement, at the beginning and during orthodontic treatment.

- It helps to identify pathologies such as cysts and tumors in bone and soft tissue.

- It provides control of the treatments and follow-up of healing.

If our patient comes to our faculty for the first time and does not have a panoramic radiograph taken within the last 6 months, a panoramic radiograph is taken from all our patients for a general evaluation of the mouth, teeth and surrounding tissues. When deemed necessary, periapical (intraoral) films can be taken from the complaint area to obtain more detailed images.

- Cone Beam Computed Tomography

In our clinic, cone beam computed tomography is also used in cases where radiographic imaging in two dimensions is insufficient, where 3-dimensional imaging of the head and neck region can be provided. Cone beam computed tomography is a radiologic method for creating a cross-sectional image of the head and neck region using X-rays. Imaging is performed with up to 90% less radiation compared to medical tomography devices used in medicine. Before the tomography, the patient should remove all metal objects in the head and neck area (earrings, hairpins, necklaces, glasses, piercings, removable prostheses, hearing aids, etc.). The patient is positioned sitting during the CT scan. The patient must remain stationary during the scan. The X-ray source rotates around the patient. The resulting images can be viewed on the computer screen and transferred to CD. If cone beam computed tomography is not performed: Three-dimensional imaging is not possible and the cause of the complaint and the success of the treatment may not be accurately determined. It is your obligation to inform your physician and the X-ray staff if you are pregnant or suspect that you may be pregnant. You must remain still during the procedure. If you move, the image may be distorted and a retake may be necessary. Cone beam computed tomography has some advantages over other X-ray examinations:

- It shows the location of bones and surrounding hard tissues very clearly.
- It helps in the differential diagnosis of cysts and tumors and provides a better evaluation of the diseases.
- It creates much more detailed images than direct radiographs.

- Taking radiographs in pregnant women

Since X-rays in dental radiology are directed only to the head and neck, the dose to the fetus is much lower than the dose received from natural sources, even when whole mouth radiographs (14 intraoral films) are taken. In spite of all this, radiography in pregnant women is taken when necessary and as few as possible. In order to prevent possible harm, the patient wears a thyroid protector made of lead- containing material and a lead apron that also

covers the abdomen.

- Photography

Photography is used to monitor certain conditions in the mouth and surrounding tissue and is used for scientific and educational purposes, after which identity information is kept confidential. Photography is expected to be useful for patient follow-up, research, scientific presentation and education.

- Vitality test

It is a test that evaluates the nervous response of the tooth to an external stimulus (cold, hot, electrical, etc.) during diagnosis and treatment planning. According to the test result, the vitality of the tooth and the treatment method to be applied to the tooth are determined. The application cannot be performed in patients with a pacemaker. If the test is not performed, the correct treatment method may not be determined. Depending on the condition of the tooth, surrounding tissue and restoration, an incorrect response may be obtained and may need to be repeated.

- Ultrasonography

It is a bedside imaging method that does not use ionizing radiation and allows the evaluation of soft tissues in the head and neck region. In the application, after the application of ultrasound gel to the body area, the probe is moved over the region. The practitioner can take sections in different planes in different planes. Tissues reflect the sound wave differently due to differences in their mechanical properties. Depending on the return time of the sound wave to the probe, tissue depth is determined. In the Doppler Ultrasonography technique, which is used to evaluate the quality and quantity of blood flow, the change in sound frequency is determined according to the direction and speed of blood flow. Patients or healthcare workers are not exposed to radiation in ultrasonography. For this reason, it is a safe diagnostic method used in children or young patients and especially in pregnant patients. If the examination cannot be performed, the pathology and its cause may not be accurately determined.

Procedures to be performed;

Digital radiography (periapical, bitewing, occlusal, panoramic, joint x-rays) ☐

Cone Beam Computed Tomography ☐

Photography ☐

Vitality test ☐

Ultrasonography ☐

CONSENT

- I am aware that Hacettepe University Faculty of Dentistry is an educational institution, that dental intern students also work here, and that students will work under the supervision of faculty members and assistants in the stages of examination, x-raying and creating a diagnosis and treatment plan.

- I consent to the participation of trainee dentists and x-ray technicians under the supervision of Oral, Dental and Maxillofacial Radiology instructors.

- If I have any systemic disease, I agree that I will inform my physician and accept the consultation procedures to be carried out by my physician.

- If I am pregnant, I agree to inform doctors and X-ray technicians of my pregnancy status.

- I agree that my anamnesis information, radiological images, photographs, examination results (pathology

report, laboratory results, etc.) may be used for diagnostic, scientific, educational or research purposes by keeping my identity information confidential.

- I have been informed that I will be directed to the relevant departments for my treatment in line with the diagnoses made in this clinic and that I can receive the information required for my treatment in detail from the relevant departments.

- I understand and accept that I have the right to refuse radiography. In this case, I understand and accept that a complete diagnosis cannot be made and that I will not hold my physician responsible for this situation.

This consent form is valid during my application to the Oral, Dental and Maxillofacial Radiology Clinic on the date I sign it. I know that I can withdraw my consent by applying in writing if I do not accept the examination and procedures in the Oral, Dental and Maxi llofacial Radiology Clinic. I accept that I have read this form and accept all the procedures to be applied to me in the light of this information.

(PLEASE SIGN BY WRITING ‘I HAVE READ AND UNDERSTOOD THIS CONSENT FORM’ IN THE SPACE BELOW)

	Name Surname	Signature	Date
Patient/Patient's Legal Representative-Degree of Proximity			
Informing Physician			
Translator (If Used)			
